# RNA modifications in the tumor microenvironment: insights into the cancer-immunity cycle and beyond

**DOI:** 10.1186/s40164-025-00648-1

**Published:** 2025-04-02

**Authors:** You-Peng Ding, Cui-Cui Liu, Ke-Da Yu

**Affiliations:** Department of Breast Surgery, Key Laboratory of Breast Cancer in Shanghai, Cancer Institute, Department of Oncology, Shanghai Medical College, Fudan University Shanghai Cancer Center, Fudan University, Shanghai, 200032 China

**Keywords:** RNA modification, Tumor microenvironment, Cancer-immunity cycle, m6A, Pseudouridylation

## Abstract

The chemical modification of biological molecules is a critical regulatory mechanism for controlling molecular functions. Although research has long focused on DNA and proteins, RNA modifications have recently attracted substantial interest with the advancement in detection technologies. In oncology, many studies have identified dysregulated RNA modifications including m6A, m1A, m5C, m7G, pseudouridylation and A to I editing, leading to disrupted downstream pathways. As the concept of the tumor microenvironment has gained prominence, studies have increasingly examined the role of RNA modifications in this context, focusing on interactions among cancer cells, immune cells, stromal cells, and other components. Here we review the RNA modifications in the tumor microenvironment through the perspective of the Cancer-Immunity Cycle. The extracellular RNA modifications including exosomes and influence of microbiome in RNA modifications are potential research questions. Additionally, RNA modifying enzymes including FTO, ALKBH5, METTL3, PUS7 are under investigation as potential biomarkers and targets for combination with immunotherapies. ADCs and mimetics of modified RNA could be potential novel drugs. This review discusses the regulatory roles of RNA modifications within the tumor microenvironment.

## Background

The tumor microenvironment (TME) is a complex and dynamic landscape, composed of diverse cellular components [1], extracellular matrix elements, and numerous signaling molecules. TME plays an important role in tumor hallmarks, such as drug resistance [2], epithelial-mesenchymal transition (EMT) [3], tumor initiation [4], progression [5] and response to immunotherapy [6]. Central to this environment are the tumor cells themselves, which orchestrate a network of interactions that support tumor growth and metastasis. Equally critical are immune cells [7], cytokines [8], cancer-associated fibroblasts [9] and various additional tissue-resident cell types, which play key roles in mediating immune responses against tumor cells. To better understand the complex interactions within the TME, the cancer-immunity cycle serves as a valuable model. Initially introduced in 2013 [10] and further refined [11] in 2023, the cancer-immunity cycle provides a conceptual framework for understanding the series of events that T cells require to generate effective anti-cancer immune responses, including release of neoantigens upon cancer cell death, antigen presentation by dendritic Cells (DCs), effector T cell priming and activation, trafficking and infiltration of T cells, and targeted cancer cell killing.

Chemical modifications are essential for precise and effective regulation of biological functions across a variety of biomolecules, including proteins, DNA, RNA, sugars, and lipids. Among these, RNA modifications exhibit particularly high diversity [12]. However, RNA modifications have long remained enigmatic due to their low abundance, structural diversity and dynamic nature [13]. With recent advancements in RNA modification detection technologies, substantial progress has been made in this field. Thus far, over 100 types of RNA modifications have been identified; each of the four RNA bases is capable of modification. These modifications occur across multiple RNA types, including messenger RNA (mRNA), transfer RNA (tRNA), ribosomal RNA (rRNA), and other non-coding RNAs, influencing processes such as RNA stability, splicing, and translation. Among the known RNA modifications, N6-methyladenosine (m6A), 5-methylcytosine (m5C), and pseudouridine are particularly noteworthy because of their prevalence and biological significance.

In the context of cancer, aberrant RNA modifications are increasingly recognized as key drivers of tumor progression and immune evasion through their roles in regulating RNA structure, gene expression, and cellular responses. By reshaping the RNA landscape within both tumor and immune cells, these modifications can extensively influence the TME and the effectiveness of immunotherapies. This review explores how specific RNA modifications, especially those involved in the cancer-immunity cycle, mediate interactions between cancer cells and the immune system. Additionally, the review examines the emerging role of RNA modification inhibitors as biomarkers and therapeutic targets, highlighting their potential to improve cancer treatment outcomes.

## RNA modifications

**Fig. 1:**
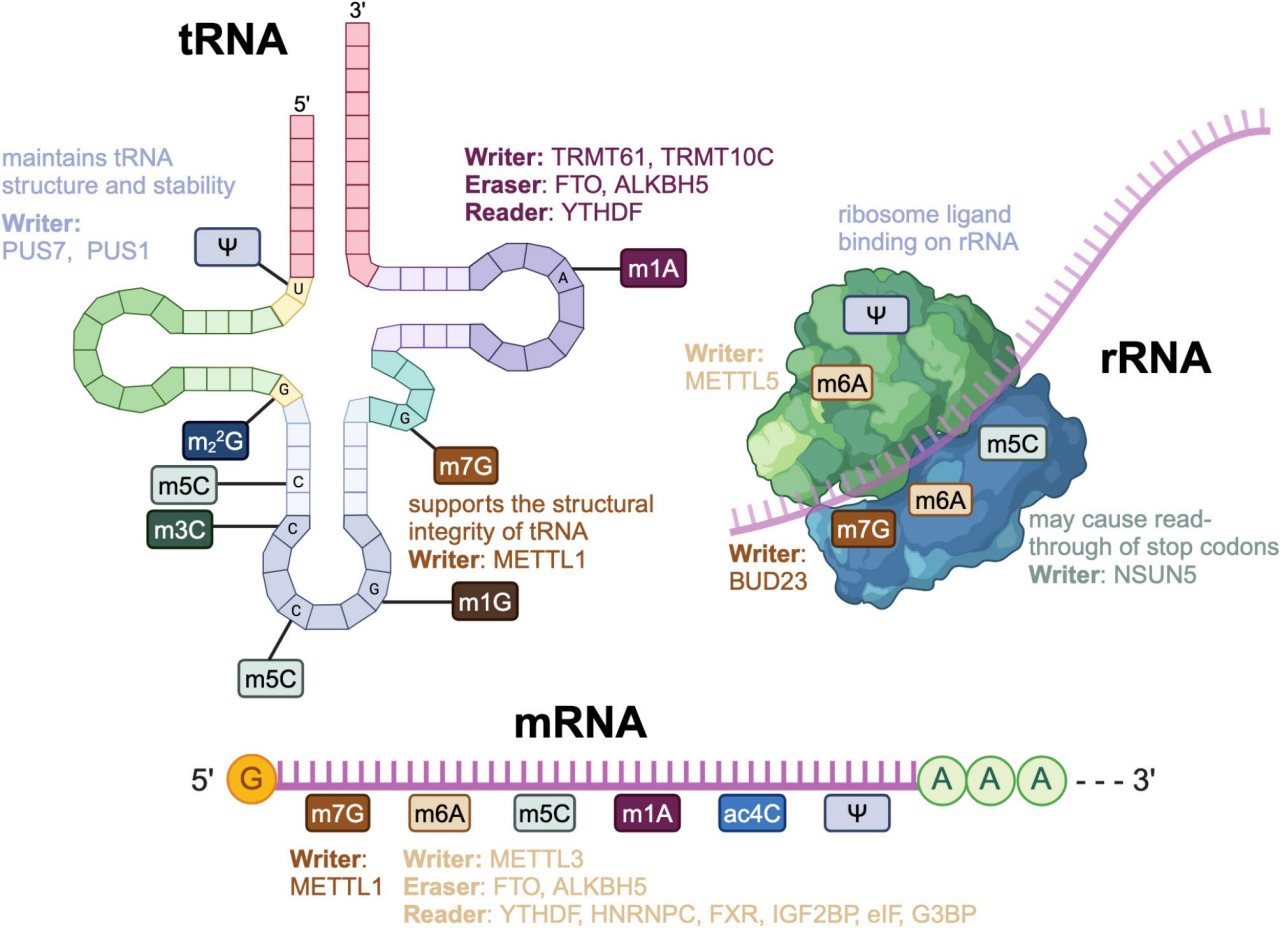
RNA modifications on different types of RNA. RNA modifications have been identified at various sites on mRNA, rRNA, and tRNA. The METTL3–METTL14 complex, METTL16, and WTAP are writers for m6A. The METTL3 is responsible for most m6A modifications on mRNA. In rRNA, TRMT112 and ZCCHC4 act as writers for m6A. m6A erasers include FTO and ALKBH5 and reader proteins include YTHDF, HNRNPC, FXR, IGF2BP, eIF and G3BP. TRMT61 is the writer of m1A in both cytoplasmic tRNA and mRNAs. In mitochondrial tRNA, m1A is catalyzed by TRMT61B and TRMT10C. Erasers of m1A include the ALKBH family and FTO. YTHDF can recognize m1A modifications. m5C can be catalyzed by NSUN5 and may cause read-through of stop codons on rRNA. m7G can be written by BUD23. PUS7 and PUS1 can write pseudouridylation, which maintains tRNA structure and stability and ribosome ligand binding on rRNA. m6A, N6-methyladenosine; m1A, N1-methyladenosine; m_2_2G, N2, N2-dimethylguanosine; m7G, N7-methylguanosine; m1G, N1-methylguanosine; m3C, 3-methylcytidine; m5C, 5-methylcytidine; Ψ, pseudouridine; ac4C, N4-acetylcytidine; PUS1: pseudouridine synthase 1; PUS7: pseudouridine synthase 7; METTL3: methyltransferase-like 3; METTL16: methyltransferase-like 16; WTAP: Wilms' tumor 1-associating protein; FTO: fat mass and obesity-associated protein; ALKBH5: AlkB homolog 5; YTHDF: YTH domain-containing family protein; HNRNPC:heterogeneous nuclear ribonucleoprotein C; FXR: fragile X-related family; IGF2BP: insulin-like growth factor 2 mRNA-binding protein; eIF: eukaryotic initiation factor; G3BP: GTPase activating protein (SH3 domain)-binding protein; NSUN5: NOP2/Sun RNA methyltransferase family member 5; BUD23: budding uninhibited by benzimidazoles 23 homolog

RNA modifications play key roles in cellular processes by modulating the stability, structure, and translational efficiency of RNA molecules. These modifications regulate gene expression post-transcriptionally, thereby influencing cell fate decisions and responses to environmental stimuli [14]. Considering their importance in cancer and immunology, this section provides an overview of key RNA modifications (Fig. 1) central to the discussion in subsequent parts of this review.

### m6A

The most prevalent and well-studied modification in eukaryotic mRNA is N6-methyladenosine (m6A) [15], which refers to the methylation of adenosine at the nitrogen-6 position. These modifications are found in mRNAs, rRNAs, long noncoding RNAs (lncRNAs), circular RNAs (circRNAs), microRNAs (miRNAs), and primary miRNAs (pri-miRNAs). Targets of m6A are primarily enriched in the coding sequence (CDS) and 3’ untranslated region (UTR) portions of mRNA at consensus sequences. m6A modifications are dynamically regulated by methyltransferases, including the methyltransferase-like (METTL)3–METTL14 complex, METTL16, and Wilms’ tumor 1-associating protein (WTAP). For example, the METTL3–METTL14 complex is responsible for most m6A modifications on mRNA; METTL3 serves as the main catalytic subunit and METTL14 functions as the RNA-binding scaffold. For m6A modifications of rRNA, the METTL5–tRNA methyltransferase activator subunit (TRMT)112 complex and zinc-finger CCHC domain-containing protein 4 (ZCCHC4) act as writers. m6A erasers include fat mass and obesity-associated protein (FTO) and AlkB homolog 5 (ALKBH5). m6A modifications are known to affect mRNA stability, splicing, and translation; specific outcomes are determined by different reader proteins, such as YTH domain-containing family protein (YTHDF)1/2/3, heterogeneous nuclear ribonucleoprotein C (HNRNPC), the fragile X-related (FXR) family, the insulin-like growth factor 2 mRNA-binding protein (IGF2BP) family, the eukaryotic initiation factor (eIF) family, and GTPase activating protein (SH3 domain)-binding proteins (G3BPs) [16]. m6A modifications can regulate tumor suppressor and oncogene expression, significantly influencing immune responses by modulating key immune signaling pathways [17].

### m1A

Another modification of adenosine, N1-methyladenosine (m1A), occurs at position 1. m1A modifications are primarily found in tRNA but also occur in rRNA, lncRNA, and mRNA [18]. The TRMT61-TRMT6 complex catalyzes m1A methylation in both cytoplasmic tRNA and mRNAs; TRMT61A serves as the catalytic subunit. In mitochondrial tRNA, m1A is catalyzed by TRMT61B and TRMT10C. Erasers of m1A include the ALKBH family and FTO. Although YTHDF1/2/3 and YTHDC1 can recognize m1A modifications through the evolutionarily conserved YTH domain, they bind with weaker affinity relative to that of m6A. Functionally, m1A stabilizes RNA structure and supports the initiation or elongation processes in tRNA, mRNA, and rRNA [19].

### m5C

Similar to adenosine, cytosine can also be modified. The 5-methylcytosine (m5C) modification appears in mRNA, tRNA, and rRNA, where it serves distinct biological roles. For example, m5C in tRNA affects structure and stability, influencing translational accuracy. When present on rRNA, m5C may cause read-through of stop codons. Through catalysis by NOP2/Sun RNA methyltransferase family member 5 (NSUN5) and other enzymes, m5C in mRNA can either promote or suppress translation, depending on the modification site [20].

### m7G

Additionally, N7-methylguanosine (m7G) is also one of the important RNA modifications [21], affecting various RNA molecules, including messenger RNA, ribosomal RNA, microRNA, and transfer RNA. In mRNA, m7G is often loacated at the mRNA cap [22], where it enhances mRNA stability and promotes efficient translation [23]. This modification also occurs in tRNA, mediated by the METTL1–WD repeat-containing protein (WDR) 4 complex, and supports the structural integrity of tRNA. In rRNA, m7G deposition is mediated by budding uninhibited by benzimidazoles 23 homolog (BUD23). Cap methylation by enzymes such as METTL1 significantly influences mRNA stability and translation, thereby affecting cellular responses [21].

### Pseudouridylation

Pseudouridylation represents the most prevalent internal post-transcriptional modification observed in stable RNAs [24, 25]. The most abundant modification in total RNA from human cells is pseudouridylation [26]. Through catalysis by pseudouridine synthases (PUSs) such as PUS7, pseudouridylation maintains tRNA structure and stability and is essential for ribosome ligand binding on rRNA. This modification may also suppress translation termination, potentially altering protein synthesis in cells [14].

### A to I editing

Another critical modification, adenosine-to-inosine (A-to-I) editing, predominantly occurs on double-stranded RNA (dsRNA) and is mediated by the adenosine deaminases acting on RNA (ADAR) enzyme family [27]. Inosines pair with cytidines rather than uridines, and the ribosome reads inosines as guanosines. Consequently, adenosine-to-inosine editing can alter protein amino acid sequences and affect cellular signaling [28].

### Detection methods

**Fig. 2:**
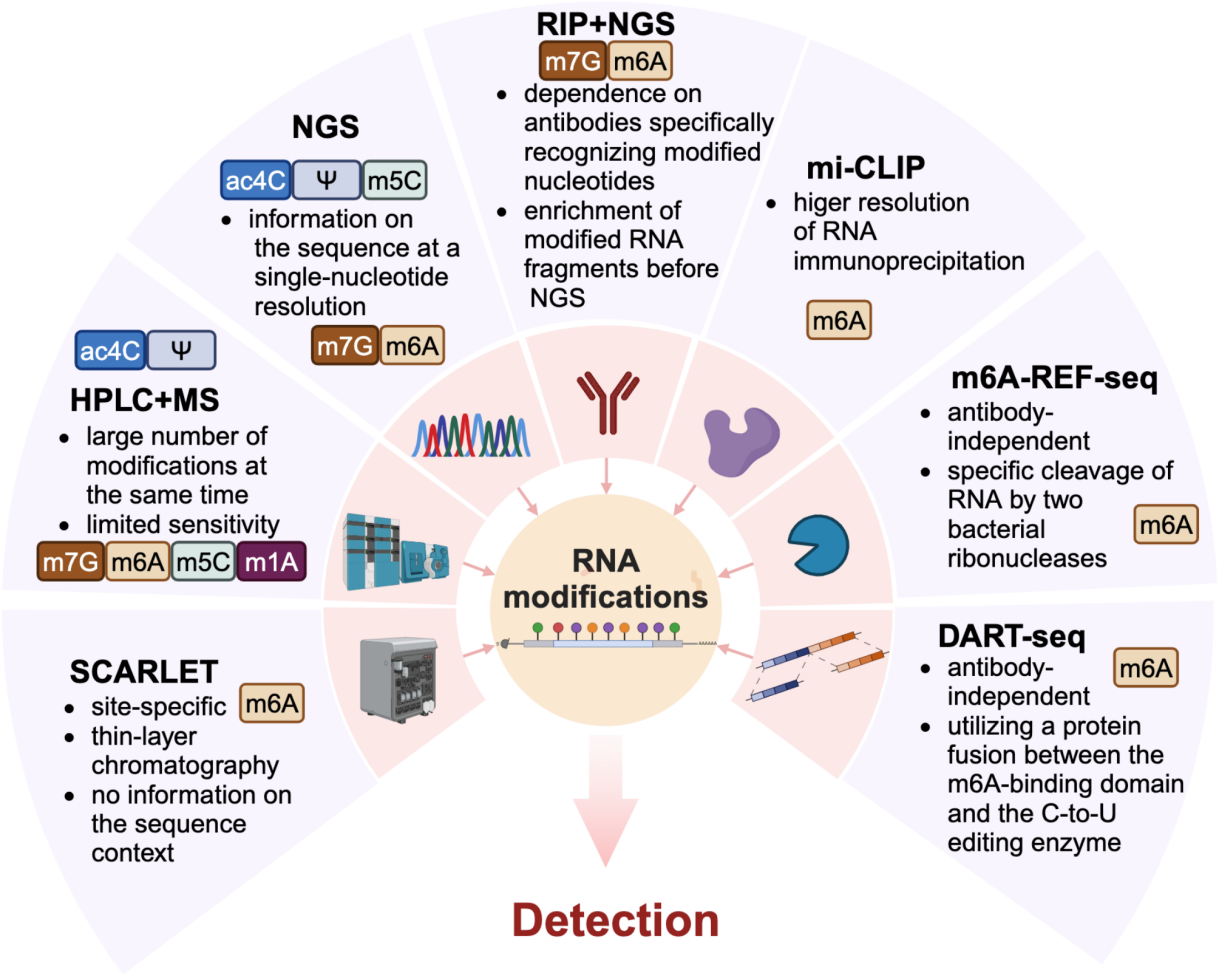
Methods for detecting RNA modifications. This figure presents multiple techniques for examining RNA modifications, and highlights the characteristics of each method as well as the modifications it can detect. SCARLET, site-specific cleavage and radioactive labelling followed by ligation-assisted extraction and thin-layer chromatography assay; HPLC, high-performance liquid chromatography; MS, mass spectrometry; NGS, next-generation sequencing; RIP, RNA immunoprecipitation; mi-CLIP, methylation–individual-nucleotide-resolution crosslinking and immunoprecipitation; m6A-REF-seq, m6A RNA endoribonuclease-facilitated sequencing; DART-seq, deamination adjacent to RNA modification targets sequencing; m6A, N6-methyladenosine; m1A, N1-methyladenosine; m7G, N7-methylguanosine; m5C, 5-methylcytidine; Ψ, pseudouridine; ac4C, N4-acetylcytidine

Advances in RNA modification research are closely linked to technological progress [29]. Numerous methodologies for RNA modification detection exist, and have been reviewed previously for comprehensive discussions and in-depth understanding [30–32]. Figure 2 highlights a selection of representative methods currently employed in the field. The following discussion will focus on an integration of mass spectrometry (MS) and single-cell technologies to explore RNA modifications. Liquid chromatography-tandem MS (LC-MS/MS) remains the standard for quantifying RNA modifications [33] due to its unparalleled sensitivity (detection limits as low as femtomolar ranges) and ability to simultaneously profile dozens of modified nucleosides, even discovering novel modifications through fragmentation patterns and retention time correlations [34]. Innovations such as the NucleicAcidSearchEngine (NASE) now enable high-throughput analysis of RNA MS data, incorporating statistical validation for reliable large-scale studies [35]. For single-cell resolution, methods like single-neuron RNA modification analysis by MS (SNRMA-MS) have emerged [36], enabling multiplexed detection of up to 16 modifications in individual cells, revealing cell-type-specific epitranscriptomic landscapes previously obscured by bulk analyses [37].

Scaling these technologies for global insights requires addressing intrinsic limitations. While MS excels in quantification, it loses sequence context upon RNA digestion, limiting its ability to map modifications to specific transcripts [38]. Nanopore sequencing, conversely, promises direct RNA sequencing with modification detection in intact molecules [39], but currently struggles with basecalling accuracy for rare modifications and requires extensive training datasets validated by orthogonal methods like MS [40]. Additionally, single-cell workflows face sensitivity challenges due to low RNA input and contamination risks, necessitating optimized microextraction protocols to isolate and analyze sub-nanogram samples [41]. Besides, despite progress in tools like NASE, computational bottlenecks still hinder scalability due to the lack of unified databases and algorithms for cross-platform data integration [42].

Future efforts may prioritize hybrid approaches, such as combining MS for stoichiometric validation, nanopore sequencing for spatial resolution and advancing computational frameworks to harmonize multi-omics datasets. Standardizing protocols for single-cell RNA modification analysis and expanding reference libraries for machine learning-driven basecalling may help to achieving scalable epitranscriptome profiling.

## Aberrant RNA modifications in the cancer-immunity cycle

**Fig. 3:**
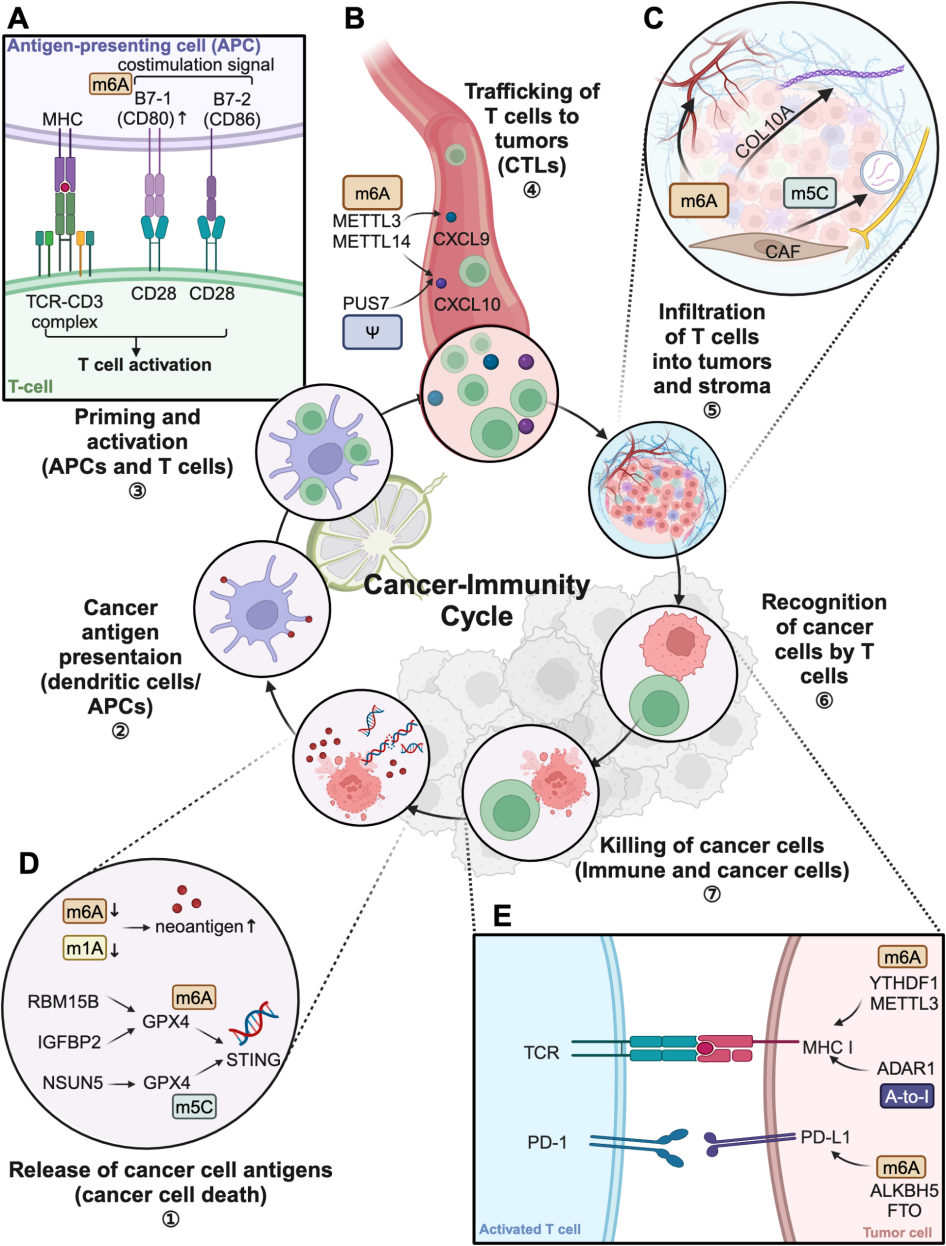
RNA modifications in the cancer-immunity cycle. The cancer-immunity cycle illustrates the steps necessary for T cells to effectively kill cancer cells. RNA modifications regulate each step of the cycle by affecting various cells and substrates within the tumor microenvironment. (**A**) Dendritic cells as well as other antigen-presenting cells (APCs) process peptide and express co-stimulatory molecules. METTL3-mediated m6A methylation of mRNA enhances the expression of co-stimulatory molecule CD80, improving antigen presentation and T cell activation. (**B**) Activated CTLs circulate throughout the bloodstream and lymphatic system, guided by chemokine. RNA modifications can influence chemokine expression, thereby affecting T cell trafficking. The m6A writers METTL3 and METTL14 suppress the expression of CXCL9 and CXCL10 in tumor cells, leading to immune exclusion in colorectal cancer. In pseudouridylation, the most abundant RNA modification in glioblastoma, PUS7 has been shown to reduce CXCL10 expression. (**C**) The infiltration of T cells within tumors are influenced by the matrix architecture which can be shaped by the accumulation of collagen. CAFs secrete collagen such as COL10A1. In LUSC, METTL3-mediated m6A modification of COL10A1 mRNA increases its stability, leading to elevated expression and increased secretion by CAFs. CAF-secreted VEGFA promotes angiogenesis and affects METTL3 expression in NSCLC cells. Besides, CAFs secrete extracellular vesicles containing PNI-associated transcripts (PIATs), relying on m5C modifications to enhance YBX1 binding to PNI-associated mRNAs to promote neural remodeling. (**D**) Tumor antigens, including neoantigens are released into the TME following cancer cell death. Low m6A score and low m1A score are linked to a higher neoantigen load. RBM15B and IGFBP2-mediated GPX4 m6A modifications, along with NSUN5-mediated GPX4 m5C modifications activate the STING pathway. (**E**) T cells rely on MHC I molecules to recognize tumor cells. In glioblastoma stem cells, the suppression of METTL3 and YTHDF2 reduces overall m6A levels, leading to increased MHC I expression. Additionally, immunosuppressive lncRNA LINC00624 stabilizes ADAR1, which edits adenosine to inosine in RNA. This modification reduces MHC I antigen presentation and decreases CD8 + T cell infiltration within the TME. In ICC, the m6A demethylase ALKBH5 reduces m6A methylation of PD-L1 mRNA, resulting in increased PD-L1 mRNA stability that suppresses T cell cytotoxic activity. In AML, inhibition of the demethylase FTO decreases the expression of immune checkpoint molecules such as PD-L1, enhancing tumor cell sensitivity to T cell-mediated cytotoxicity. m6A, N6-methyladenosine; m1A, N1-methyladenosine; m5C, 5-methylcytidine; Ψ, pseudouridine; APC, antigen-presenting cell; CTL, cytotoxic T lymphocyte; CAF, cancer-associated fibroblast; TCR, T cell receptor; MHC I, major histocompatibility complex I; PD-1, programmed cell death 1; PD-L1, programmed cell death-ligand 1; APC, antigen-presenting cell; CTL, cytotoxic T lymphocyte

The Cancer-Immunity Cycle outlines the process by which T cells recognize and destroy cancer cells; it also describes how tumors develop mechanisms to evade this destruction. Extensive research has explored the mechanisms underlying each step of the cycle, revealing a pivotal role for RNA modifications in regulating its dynamics. This section examines the influence of RNA modifications at each step (Fig. 3), emphasizing their effects on the delicate balance between promoting and inhibiting tumor progression and immune response.

### Release of cancer cell antigens

The cancer-immunity cycle begins with the release of tumor antigens, including neoantigens, into the TME following cancer cell death. These neoantigens are generated from mutations, alterations in RNA splicing, RNA modifications, the expression of viral open reading frames, including de novo viral infections, the activation of endogenous retroviruses (ERVs) and other transposable elements [43]. RNA modifications can alter neoantigens, thus affecting tumor cell exposure to host immune surveillance. For instance, a low m6A score—determined by the expression patterns of m6A-associated genes—is linked to a higher neoantigen load, immune activation, an inflamed TME, and an improved response to anti-programmed cell death protein (PD)-1/ligand (L)1 immunotherapy in gastric and head and neck cancers [44]. Conversely, in prostate cancer, elevated levels of m6A regulators, such as the m6A writer METTL14, are associated with low tumor heterogeneity and reduced neoantigen levels [45]. Studies on bladder cancer have shown similar results with lncRNAs; specifically, high m6A modification levels in lncRNAs are associated with low neoantigen loads and poor responses to immunotherapy [46]. Although m6A modifications have robust effects, other types of methylation modifications, such as m1A, display comparable outcomes. A low m1A score is associated with a high neoantigen burden, which leads to increased proliferation of cluster of differentiation (CD)8 + T effector cells, enhanced responses to anti-PD-L1 immunotherapy, and prolonged survival in colon cancer [47].

During immunogenic cell death (ICD), cancer cells release various intracellular components, including double-stranded DNA (dsDNA), which activates the cGMP-AMP synthase (cGAS)-stimulator of interferon genes (STING) pathway [48]. Activation of the STING pathway is essential for initiating the cancer-immunity cycle because it promotes an inflammatory environment and recruits immune cells to the tumor site, expanding the immune response [49]. RNA modifications can influence the STING pathway, thereby affecting the TME. For instance, RBM15B- and IGFBP2-mediated GPX4 m6A modifications, along with NSUN5-mediated GPX4 m5C modifications, affect redox homeostasis, activate the STING pathway, and enhance anticancer immunity in colorectal adenocarcinoma (COAD) [50]. Additionally, m6A methylation enhances cGAS-STING recognition of cytosolic dsDNA in a sequence-specific manner; it is immunostimulatory in certain motifs and immunosuppressive in others [51].

### Cancer antigen presentation and T cell activation

Dendritic cells (DCs) as well as other antigen-presenting cells (APCs) process peptide and express co-stimulatory molecules, thus playing pivotal roles in the TME [52]. DC has subset diversity, including cDC1, cDC2, pDCs and they have distinct roles in tumor immunity [53]. For instance, cDC1s specialize in cross-presenting tumor antigens to CD8 + T cells via MHC-I. They orchestrate immune responses through dynamic interactions with naive T cells [54].

RNA modifications primarily impact DC antigen presentation, particularly through peptide processing. In melanoma and colorectal cancer, the m6A reader YTHDF1 reduces the ability of DCs to present tumor neoantigens to T cells. During antigen processing, cathepsins degrade proteins into smaller peptides for presentation via MHC molecules. YTHDF1 in DCs enhances the translation of cathepsin transcripts, increasing lysosomal protease activity and resulting in excessive protein degradation, inefficient antigen presentation, and limited immune recognition [55]. RNA modifications also play a critical role in the phenotypic and functional maturation of DCs. METTL3-mediated m6A methylation of mRNA significantly enhances the expression of co-stimulatory molecules CD40 and CD80 (B7.1), thereby improving antigen presentation [56]. Additionally, TNF-α, another essential stimulatory factor in antigen presentation, is regulated by both METTL3 [57] and the m6A demethylase ALKBH5 [58].

In addition to their effects on antigen presentation, RNA modifications in DCs strongly influence T cell activation. As previously noted, METTL3-mediated m6A modification enhances the translation of co-stimulatory molecules such as CD40 and CD80 (B7.1) in DCs, promoting T cell activation both in vitro and in vivo [56]. Furthermore, METTL3-mediated m6A modification of Tirap, a Toll-like receptor (TLR)4 signaling adaptor, enhances the TLR4/nuclear factor kappa-light-chain-enhancer of activated B cells (NF-κB) signaling pathway. This modification increases the secretion of pro-inflammatory cytokines, including interleukin (IL)-12, which stimulates T cell activation and differentiation from naive CD4 + T cells into Th1 cells. IL-12 also plays a role in stimulating CD8 + cytotoxic T lymphocytes (CTLs), increasing their proliferation and cytotoxic activity [56]. However, the full implications of these RNA modifications on T cell priming within the TME require further investigation.

### Trafficking of T cells to tumors

Activated CTLs circulate throughout the bloodstream and lymphatic system. Under the influence of chemokines, which can be secreted by tumor cells or other cells within the TME, these CTLs migrate toward tumor sites. Chemokines bind to receptors on T cells, guiding them toward areas with higher chemokine concentrations. The CXCR3-CXCL9/10 axis, in particular, plays a critical role in this process as a strong stimulatory factor [59]. RNA modifications can influence chemokine expression, thereby affecting T cell trafficking. For example, in tumor cells, the m6A writers METTL3 and METTL14 suppress the expression of chemokines CXCL9 and CXCL10, leading to immune exclusion. Conversely, deletion of METTL3 or METTL14 in tumors increases the expression of signal transducer and activator of transcription (STAT)1 and interferon regulatory factor (IRF)1; the resulting elevated secretion of CXCL9 and CXCL10 recruits CD8 + T cells and natural killer (NK) cells into the TME in colorectal cancer [60]. Regarding RNA modification of CXCR3, studies indicate that the demethylase FTO can regulate the m6A methylation levels of CXCR3 [61]; however, its specific role in tumors remains under investigation. In the context of pseudouridylation, the most abundant RNA modification in glioblastoma, PUS7 has been shown to reduce CXCL10 expression. This modification by PUS7 is associated with worse survival outcomes in patients with glioblastoma [62].

### Infiltration of T cells into tumors

#### Accumulation of activated T cells in and through the stroma

The infiltration, movement, and retention of T cells within tumors are influenced by the matrix architecture of the TME, which is largely shaped by cancer-associated fibroblasts (CAFs). Of the three CAF subtypes identified in solid tumors, myofibroblastic CAFs are especially prominent for their ability to produce extensive extracellular matrix components and other fibrosis-associated molecules. These elements substantially impact immune cell infiltration, including T cell accumulation, as well as cancer cell invasiveness and organ stiffness. RNA modifications play a crucial role in regulating CAF activity within the TME, specifically affecting T cell infiltration and accumulation in tumors.

CAFs exhibit distinct RNA modification patterns essential for their function, especially in modulating the TME to either facilitate or hinder T cell infiltration. For instance, in conjunctival melanoma, single-cell transcriptome analysis revealed that the RNA demethylase FTO is specifically upregulated in CAFs. This upregulation of FTO promotes CAF-mediated angiogenesis by demethylating m6A modifications on vascular endothelial growth factor (VEGF)A and early growth response (EGR)1 transcripts. This process disrupts the YTHDF2-dependent mRNA decay pathway, stabilizing and upregulating VEGFA and EGR1 mRNA, ultimately leading to enhanced angiogenesis [63]. This enhanced angiogenesis can remodel the tumor vasculature and stroma, thereby influencing T cell infiltration into the tumor site.

In non-small cell lung cancer (NSCLC), CAF-secreted VEGFA promotes angiogenesis and affects RNA modifications within tumor cells. CAF-derived VEGFA affects METTL3 expression in NSCLC cells, which is associated with poor clinical prognosis. This upregulation increases m6A modifications of Ras-related C3 botulinum toxin substrate 3 (RAC3) mRNA in NSCLC cells, enhancing the stability and translation of RAC3, activating the protein kinase B (AKT)/NF-κB pathway, and subsequently promoting NSCLC cell migration and invasion [64]. These alterations in tumor and stromal cells may create a microenvironment that either facilitates or suppresses T cell infiltration, thereby impacting the anti-tumor immune response.

Another well-established function of CAFs is the secretion of collagen, such as COL10A1, which promotes tumor proliferation and inhibits apoptosis. RNA modifications also play a role in this process. In lung squamous cell carcinoma (LUSC), METTL3-mediated m6A modification of COL10A1 mRNA in CAFs increases its stability, leading to elevated COL10A1 expression and increased secretion of COL10A1 by CAFs [65]. The accumulation of collagen and other extracellular matrix components can form physical barriers that impede T cell penetration into tumors.

There are links between the nervous system and the induction of hallmark capabilities, revealing neurons and axons as hallmark-inducing constituents of the TME [66]. Additionally, RNA modifications have been implicated in CAF-driven neural remodeling within the TME, indirectly affecting T cell infiltration by altering the tumor’s structural and functional landscapes. Clinical data, such as endoscopic ultrasonographic elasticity scoring, have shown a strong correlation between CAFs and perineural invasion (PNI) in pancreatic cancer. Mechanistic analyses have shown that CAFs secrete extracellular vesicles containing PNI-associated transcripts (PIATs), a process that relies on m5C modifications to enhance YBX1 binding to PNI-associated mRNAs, thereby promoting neural remodeling [67].

RNA modifications can also enhance the invasive capacity of CAFs, thus altering the tumor stroma and collagens affecting T cell movement. In colorectal cancer, adenosine-to-inosine (A-to-I) RNA editing of antizyme inhibitor 1 (AZIN1) by ADAR1 has been shown to increase CAF invasiveness, highlighting the importance of RNA editing in modulating CAF functions [68].

Overall, RNA modifications are essential for regulating CAF functions within the TME, as well as influencing T cell infiltration, accumulation, and effectiveness within the tumor stroma.

#### Interactions with immune cells

In the TME, T cells interact with various immune cells, including those within tumor-associated lymphoid aggregates and tertiary lymphoid structures (TLSs). Myeloid cells, including macrophages [69] and myeloid-derived suppressor cells (MDSCs), are the most abundant non-cancerous cell types in solid tumors, comprising nearly half of all cells in the TME. Numerous studies have linked tumor-associated myeloid cells to poor prognoses and limited responses to anti-cancer therapies. RNA modifications play key roles in these tumor-associated myeloid cells.

In 2019, researchers discovered that RNA modifications could influence macrophage behavior. For instance, the m6A reader YTHDF2 regulates the inflammatory response in lipopolysaccharide (LPS)-stimulated macrophages by reducing the stability and expression of mitogen-activated protein kinase kinase 4 (MAP2K4), which inactivates the MAPK and NF-κB signaling pathways. This reduction decreases the expression of pro-inflammatory cytokines such as IL-6, tumor necrosis factor (TNF)-α, IL-1β, and IL-12, ultimately diminishing the inflammatory response in LPS-stimulated macrophages [70]. A recent study explored the relationship between RNA modifications and macrophage polarization. The N6-methyladenosine demethylase FTO promotes activation of both M1 and M2 macrophages. Through YTHDF2 involvement, FTO maintains the stability of STAT1 mRNA in M1-polarized macrophages and peroxisome proliferator-activated receptor (PPAR)-γ mRNA in M2-polarized macrophages. This regulation activates the NF-κB signaling pathway via phosphorylation of IKKα/β, IκBα, and p65, promoting both M1 and M2 macrophage polarization [71].

##### Tumor-associated macrophages

Research within the TME has shown that RNA modifications can affect tumor-associated macrophages (TAMs), thereby influencing T cell infiltration. For example, the deletion of Mettl3 in mouse myeloid cells promotes tumor growth and metastasis in vivo by increasing regulatory T cell (Treg) infiltration into tumors and reducing PD-1 checkpoint blockade efficacy in Mettl3-deficient mice [72]. Additionally, METTL3 and the reader HNRNPA2B1 control the maturation of miR-146b by regulating its m6A modification, which promotes M2-TAM polarization via the phosphoinositide 3-kinase (PI3K)/AKT signaling pathway, reducing T cell infiltration, increasing immunosuppression, and ultimately advancing tumor progression in colorectal cancer [73]. Another study focused on the role of METTL14 in TAMs, revealing that its loss promotes CD8 + T cell dysfunction and tumor growth. Macrophage-specific knockout of METTL14 decreases m6A abundance on, and increases levels of, transcripts encoding Ebi3—a cytokine subunit that drives CD8 + T cell differentiation along a dysfunctional trajectory, impairing their tumor-eliminating capabilities. In the clinical setting, METTL14-m6A levels are negatively correlated with dysfunctional T cell levels in colorectal cancer patients, highlighting the clinical relevance of this regulatory pathway [74].

Because TAMs are embedded within the TME, they are also influenced by RNA modifications in tumor cells. Studies in hepatocellular carcinoma (HCC) and NSCLC have shown that tumor cell RNA modifications, such as those mediated by the N6-methyladenosine (m6A) demethylase ALKBH5, impact TAMs by regulating chemokine expression and thus affecting macrophage recruitment. Mechanistic analyses have shown that ALKBH5 upregulates MAP3K8 in an m6A-dependent manner, activating the c-Jun N-terminal kinase (JNK) and extracellular signal-regulated kinase (ERK) pathways and increasing IL-8 expression to promote macrophage recruitment in HCC [75]. In NSCLC, ALKBH5 expression within tumor cells enhances the secretion of CCL2 and CXCL10, thereby recruiting PD-L1 + TAMs and promoting macrophage polarization toward the M2 phenotype [76]. Clinical data indicate that high ALKBH5 expression in these cancers is associated with poor prognosis and shows correlations with increased macrophage infiltration and M2 polarization. Moreover, tumor cell RNA modifications can influence the phagocytic function of macrophages. For instance, high expression of the RNA N6-adenosine methyltransferase METTL14 in tumor cells increases the proportion of PD-1 + TAMs, which lack the ability to engulf tumor cells, thus inhibiting macrophage recognition and phagocytosis of tumor cells [77].

##### MDSCs

In the TME, MDSCs play a crucial role in interactions among immune cells; RNA modifications have been identified as important regulators of their function. For instance, recent studies demonstrated that deletion of the m6A demethylase ALKBH5 sensitizes tumors to cancer immunotherapy by reducing the accumulation of MDSCs and Tregs within the microenvironment [78].

Research into tRNA modifications, particularly involving METTL1 (an enzyme responsible for m7G tRNA modification), has also progressed. In advanced intrahepatic cholangiocarcinoma (ICC), polymorphonuclear MDSCs (PMN-MDSCs) are significantly enriched and correlated with METTL1 expression. In humans, CXCL8 and in mice, Cxcl5, are key targets of METTL1, facilitating the accumulation of PMN-MDSCs and promoting ICC progression [79]. Similar findings have been observed in HCC after insufficient radiofrequency ablation (RFA), a critical treatment for HCC. METTL1 expression is increased in recurrent HCC post-RFA. This heat-induced upregulation enhances transforming growth factor (TGF)-β2 translation, which recruits MDSCs and reduces the CD8 + T cell population [80].

#### Maintenance of effector state and function

The ability of T cells to maintain their effector state and function within the TME is influenced by environmental factors such as oxygen levels, pH, and nutrient availability. These conditions can significantly impact T cell activation, proliferation, and overall effectiveness in targeting tumor cells [81]. 

Recent discoveries underscore the critical role of RNA modifications in regulating immune functions within the TME. There is evidence that m6A modifications support the suppressive functions of Tregs [82]. In prostate cancer, the m6A reader HNRNPC suppresses the TME by activating Treg cells, thus promoting cancer progression [83]. Additionally, T cell homeostasis can be regulated through m6A-modified mRNA targeting the IL-7/STAT5/suppressor of cytokine signaling (SOCS) pathways [84].

The TME often exists in a hypoxic state. the m6A demethylase ALKBH5 contributes to this state by inducing paraspeckle assembly and IL-8 secretion, fostering an immunosuppressive environment [85].

Furthermore, because of metabolic abnormalities and vascular disarray, lactate accumulation within the TME decreases pH. ALKBH5 modulates the response to anti-PD-1 therapy by influencing lactate levels and the accumulation of suppressive immune cells in the TME [78].

### T cell recognition of cancer cells

For effective recognition and targeting of tumor cells, T cells rely on the functionality of MHC I molecules, which are essential for presenting tumor-specific antigens on cell surfaces. RNA modifications that modulate MHC I expression significantly influence this recognition process, thereby impacting the efficacies of immune responses and immunotherapies [86]. 

The m6A reader protein YTHDF1 plays a key role in reducing MHC I expression in tumor cells. Mechanistic analyses have revealed that YTHDF1 enhances the translation of lysosomal genes, promoting lysosomal proteolysis of MHC I and antigens; these processes facilitate immune evasion and resistance to immune checkpoint inhibitors (ICIs). Conversely, YTHDF1 deficiency can convert immunologically “cold” tumors into “hot” tumors, making them more responsive to ICI therapies and thus enhancing therapeutic efficacy [87]. In glioblastoma stem cells, the suppression of METTL3 and YTHDF2 reduces overall m6A levels, leading to increased interferon signaling and subsequent MHC I expression. This modulation enhances immunotherapy effectiveness by improving the immune system’s ability to recognize and target cancer cells [88]. Additionally, immunosuppressive lncRNAs, such as LINC00624, promote tumor progression and therapy resistance by stabilizing ADAR1, which edits adenosine (A) to inosine (I) in RNA. This modification of the double-stranded structure of LINC00624 reduces MHC I antigen presentation and decreases CD8 + T cell infiltration within the TME [89]. These insights underline the complex interplay between RNA modifications and immune surveillance mechanisms. By modulating the expression and functionality of MHC I, RNA modifications can either hinder or enhance the ability of T cells to recognize and eliminate tumor cells, with profound implications for the development of more effective cancer immunotherapies.

### Killing of cancer cells

The elimination of cancer cells represents the culmination of the cancer-immunity cycle, a critical process in which immune cells (e.g., CTLs) recognize and destroy malignant cells [90]. A central mechanism in this process is the interaction between the PD-1 receptor on T cells and its ligand PD-L1 on tumor cells. The binding of PD-L1 to PD-1 inhibits T cell activation, allowing cancer cells to evade immune-mediated destruction—a process known as immune escape [91]. 

Recent studies have shown that RNA modifications can substantially influence this interaction, impacting the efficacy of the cancer-immunity cycle. RNA modifications, particularly m6A methylation, play pivotal roles in regulating the expression of immune checkpoint molecules such as PD-L1, thereby modulating the immune escape abilities of tumor cells [92]. 

In ICC, the m6A demethylase ALKBH5 directly targets PD-L1 mRNA, influencing its stability and expression. ALKBH5 interaction with PD-L1 mRNA reduces m6A methylation within the 3’ UTR portion of that mRNA. This reduction prevents recognition by the m6A reader YTHDF2, resulting in increased PD-L1 mRNA stability that suppresses T cell cytotoxic activity and facilitates tumor immune evasion [92]. In acute myeloid leukemia, inhibition of the demethylase FTO decreases the expression of immune checkpoint molecules such as PD-L1 and leukocyte immunoglobulin-like receptor (LILR)B4, enhancing tumor cell sensitivity to T cell-mediated cytotoxicity. These findings highlight the potential of RNA demethylase targeting to strengthen the immune response against cancer cells [93]. Conversely, in melanoma cells, PD-1 expression is positively regulated by FTO. Under metabolic stress or starvation conditions, melanoma cells upregulate FTO, which reduces m6A deposition on PD-1, CXCR4, and SRY-box transcription factor (SOX)10 mRNAs. This increase in methylation accelerates mRNA decay via YTHDF2, thereby preventing tumor growth and potentially improving responses to anti-PD-1 therapies [94].

## Future directions in RNAs modifications within the TME

### Extracellular RNA modifications

The non-cellular components of the TME, such as extracellular vesicles (e.g., exosomes), metabolites, and extracellular matrix, are increasingly recognized as critical regulators of tumor progression [95]. Among these, RNA modifications and their carriers—particularly exosome-derived RNAs—play pivotal roles in shaping TME [96]. RNA modifications can appear both intracellularly and extracellularly, but the estimation of abundance is challenging. Extracellularly, exosomal RNA modifications exist at low concentrations and are masked by non-tumor-derived vesicles, necessitating ultra-sensitive techniques [97]. Additionally, exosome isolation methods like ultracentrifugation and microfluidics often introduce biases, potentially altering RNA modification profiles [98]. Exosome-mediated exchange of modified RNAs could be a key mechanism of TME reprogramming. In colon cancer, m6A modification genes in exosomes significantly influence the TME, where a low m6A-related exosomal gene score (MREGS) is associated with enhanced survival, immune activation, and a better response to anti-PD-L1 immunotherapy, while a high MREGS correlates with increased stromal activation, heightened innate immune cell activity, and poorer survival outcomes [99]. 

More researches could focus on RNA modifications serving as molecular bridges linking intracellular epitranscriptomic regulation to extracellular TME crosstalk, with exosomes acting as critical vehicles. Overcoming technical limitations in quantifying these modifications will be essential for harnessing their clinical potential.

### Influences of microbiome in RNAs modifications

Numerous studies have demonstrated the significant role of the microbiome in human cancer [100]. The gut microbiome, recognized for its influence on the host’s immune system and metabolism, impacts tumor progression [101]. Recent insights also highlight the effects of the local tumor microbiome on tumor growth and treatment outcomes [102]. Intratumoral microbiomes have been identified in various cancers including pancreatic, colorectal [103], liver [104], esophageal, breast, and lung cancers [105].

As research progresses, the perspective of RNA modification increasingly contributes to our understanding of how the microbiome affects the host. In 2019, a study revealed that the presence of microbiota correlates with low m6A levels, displaying tissue-specificity, particularly in the brain [106]. Specifically, among the brain, intestine and liver tissues, the largest effect is present in the brain, which is associated with upregulation of both m6A writer and eraser proteins, so that the brain tissue may be more sensitive to adjust the m6A under the influence of the microbiome compared with other tissues [106]. By 2024, using nanopore direct RNA sequencing, the microbiome’s role in modifying various RNA modifications, including m6A, m5C, and Ψ, as well as affecting isoform generation, poly(A) tail length, and transcript abundance, was further elucidated not only in the brain but also in the cecum [107].

A study within the context of tumor microenvironment has shown that Fusobacterium nucleatum, which is enriched in colorectal cancer tissues, was found to upregulate PD-L1 protein expression and contribute to an immunosuppressive tumor environment through mechanisms involving RNA modifications [108]. Mechanically, m6A-modified IFIT1 mediated the F. nucleatum-induced upregulation of PD-L1, with alterations in m6A levels in IFIT1 mRNA’s 3’UTR triggered by F. nucleatum treatment [108].

Currently, research into the impact of microbiome on RNA modifications within the tumor microenvironment remains limited. However, the critical role of microbiota in cancer and the diversity and significance of RNA modifications suggest promising prospects for future studies.

## From bench to bedside

The findings above highlight the complex roles of RNA modifications in regulating the expression of critical immune checkpoint molecules and modulating various immune cells, underscoring the potential of targeting these modifications to enhance cancer immunotherapy effectiveness. Approaches that manipulate RNA modifications may shift the balance of the cancer-immunity cycle toward more efficient immune-mediated tumor cell destruction.

### Prognosis and biomarkers

Research progress has substantially enhanced our understanding of RNA modification patterns and scoring systems. These biomarkers extend beyond predictions of overall prognoses; they also reveal essential characteristics of the TME and enable predictions of responses to immune therapies. Table 1 below presents a range of RNA modifications across different cancer types established in previous researches, highlighting their associations with disease prognosis and immune cell infiltration in the TME.

However, the observed differences in RNA modification-clinical correlations across tumor types likely stem from a combination of biological heterogeneity and technical variability.

Biologically, distinct TMEs exhibit unique cellular compositions including immune/stromal cell ratios and metabolic states that shape RNA modification dynamics. For example, ADAR1’s role as a prognostic biomarker in gastric cancer [109] versus prostate cancer [110] may be caused by differences in immune infiltration or tumor-intrinsic pathways (e.g. IFN-γ signaling vs. androgen receptor activity) [111]. Mechanical studies are needed to answer these questions.

Technical limitations further complicate cross-study comparisons. For instance, the scoring systems vary, including e.g., Writer-Score [112], WM-Score [113], RMW Score [114], etc. Besides, different RNA detection technologies can also artificially inflate discrepancies. Additionally, differences in sample preservation (e.g., FFPE vs. fresh tissues) or sequencing platforms (e.g., Illumina vs. nanopore) can alter RNA modification detection sensitivity, particularly for low-abundance marks.

Future studies are needed for standard multi-omics workflows that integrate single-cell RNA modification profiling with spatial transcriptomics to mark cell type-specific effects. Besides, unified scoring systems adjusted for tumor purity and immune/stromal content should be established to harmonize cross-cohort data.

By reconciling biological complexity with technical rigor, RNA modification biomarkers may achieve broader clinical utility.

### Therapeutic targets

#### RNA modifying enzymes

In the context of recent research advances, RNA modifying enzymes are increasingly recognized as biomarkers, as well as viable therapeutic targets in cancer treatment. Inhibitors targeting specific RNA modifications have shown potential to enhance the efficacy of cancer immunotherapies, highlighting their dual roles in tumor characterization and treatment.

For instance, inhibition of Alkbh5, a demethylase involved in RNA modifications, significantly enhances cancer immunotherapy effectiveness. Studies have shown that a small-molecule Alkbh5 inhibitor can amplify the immune response against tumors, leading to improved therapeutic outcomes [78]. Additionally, co-blockade of METTL1 and its downstream chemokine pathways increases the efficacy of anti-PD-1 therapies in preclinical models of ICC [79]. These findings suggest that targeting RNA methyltransferases and their associated signaling pathways can synergistically enhance immune checkpoint blockade therapies. Moreover, N-acetyltransferase 10 (NAT10) [115], which plays a role in N4-acetylcytidine (ac4C) mRNA modification, promotes metastasis in head and neck squamous cell carcinoma and remodels the TME through the MAPK/ERK signaling pathway. Specific inhibitors such as remodelin can reshape the TME, enhancing CD8 + T cell and Treg recruitment and thus improving immune-mediated tumor suppression [116].

Furthermore, the small molecule cucurbitacin B (CuB), which directly targets IGF2BP1, has shown the ability to activate immune cell infiltration—including CD4 + and CD8 + T cells, CD56 + NK cells, and F4/80 + macrophages—and reduce PD-L1 expression in HCC [117]. These results demonstrate the potential of RNA-binding protein inhibitors to modulate the immune environment and improve responses to immunotherapeutic agents.

Table 2 provides additional information on RNA modification inhibitors and their potential to enhance immune-based cancer therapies, detailing various compounds, their targets, and effects across different tumor and immunotherapy contexts.

#### Mimetics of modified RNA

Currently, the therapeutic targets are focused on RNA modifying enzyme inhibitors. However, the use of mimetics of modified RNAs could serve as combinatorial therapeutic agents to reshape the tumor microenvironment and enhance immune responses, offering new avenues for cancer treatment.

Additionally, modifications such as pseudouridylation enhance the stability, translational efficiency, and T cell-stimulatory capacity of mRNA vaccines [118] and DC vaccines [119]. These modifications improve the expression of encoded antigens in DCs, increasing the density of antigen-specific peptide/MHC complexes and strengthening the ability of these DCs to stimulate and expand specific CD4 + and CD8 + T cells.

#### ADCs as potential drugs

While current studies on RNA-modifying enzyme inhibitors are characterized in the context of a given cancer indication, emerging evidence underscores the importance of reconciling their activity upon different immune cell populations to fully unravel their biological complexity and therapeutic potential.

For instance, targeted delivery strategies, such as antibody-drug conjugates (ADCs), may help to confine inhibitor activity to tumor cells while sparing immune cells from unintended suppression [120].

To address the need for pre-clinical frameworks that reconcile RNA-modifying enzyme inhibitor activity across tumor and immune compartments, a multi-modal evaluation strategy is essential.

First, target validation should integrate single-cell RNA-seq and spatial transcriptomics to map the expression of the enzyme (e.g. METTL3, ADAR1) across tumor and immune subsets within the tumor microenvironment (TME), ensuring tumor-specificity and identifying immune cell vulnerabilities [121].

Second, delivery optimization via antibody-drug conjugates (ADCs) [122] could exploit tumor-specific antigens (e.g. HER2 [123], TROP-2 [124]) to localize inhibitors (e.g. FTO or ALKBH5 inhibitors) to cancer cells, leveraging stable linkers and high drug-antibody ratios (DAR) to minimize off-target immune suppression.

Third, preclinical models must include immune-competent systems, such as humanized mice or co-cultures of tumor organoids with autologous immune cells, to assess inhibitor effects on immune checkpoint including PD-L1, cytokine secretion, and T/NK cell cytotoxicity [125].

Finally, safety screens must evaluate systemic and immune toxicity, including myeloid suppression or autoimmune activation, using flow cytometry and cytokine arrays.

This framework aims to prioritize tumor-selective delivery while capturing the TME’s complexity, hoping for the development of RNA-modifying therapies that synergize with immunotherapy.

## Conclusions

Studies of RNA modifications have revealed their pivotal role in the complex interactions between cancer cells and the immune system within the TME, influencing each step of the cancer-immunity cycle. This review has highlighted how RNA modifications regulate immune surveillance, antigen presentation, and overall tumor immunity, strongly influencing therapeutic responses. The identification of RNA modifications as both biomarkers and therapeutic targets underscores their potential to transform cancer treatment strategies.

RNA modifications have multifaceted roles within the TME. For instance, both in vitro and in vivo studies have demonstrated that tumor-derived ALKBH5 can suppress T cell proliferation and cytotoxicity by maintaining PD-L1 expression on tumor cells [92]. However, ALKBH5 also promotes PD-L1 expression on monocytes/macrophages and decreases MDSC infiltration [92]. Ultimately, patient samples showing strong nuclear expression patterns of ALKBH5 tend to respond better to anti-PD-1 immunotherapy [92]. This finding highlights the complex roles of RNA modifications within the TME and underscores the need for further investigation to fully understand their implications in cancer biology and treatment. Future studies could explore the role of RNA modifications in more specific cell types.

Continued research into the molecular mechanisms of RNA modifications is expected to facilitate the development of more refined and personalized therapeutic approaches, ultimately improving outcomes for cancer patients. The integration of these novel molecular insights into clinical practice will likely expand the therapeutic landscape, leading to more effective interventions against cancer.

**Table 1:** Comprehensive overview of RNA modifications as biomarkers in cancer

RNA modification	biomarkers	cancer type	function	references
pseudouridylation	PUS1	hepatocellular carcinoma	tumor grade, prognosis	[126]
	PUS7	glioblastoma	prognosis	[62]
adenosine-to-inosine	ADAR1	gastric cancer	prognosis	[109]
	ADAR1	prostate cancer	prognosis	[110]
	ADAR1	endometrial cancer	prognosis	[127]
	ADAR1	lung adenocarcinoma	prognosis	[128]
	ADAR1	colorectal cancer	prognosis	[129]
m6A	scoring	esophageal cancer	prognosis, infiltration of immune cells in TME	[130]
	scoring	renal papillary cell carcinoma	prognosis, infiltration of immune cells in TME	[131]
m5C	m5C RNA modification-related gene index (M5CRMRGI)	clear cell renal cell carcinoma	prognosis, infiltration of immune cells in TME	[132]
	scoring	lung adenocarcinoma	prognosis, infiltration of immune cells in TME	[133]
	scoring	prostate adenocarcinoma	infiltration of immune cells in TME	[134]
m7G	WDR4	breast cancer	immunotherapy resistance	[135]
	scoring	glioblastoma	infiltration of immune cells in TME	[136]
m6A/ m1A/ alternative polyadenylation/ adenosine-to-inosine	scoring	non-small cell lung cancer	prognosis, immunotherapy resistance	[112]
	scoring	colorectal cancer	prognosis, infiltration of immune cells in TME	[113]
	scoring	pancreatic cancer	prognosis, infiltration of immune cells in TME	[137]
	scoring	ovarian cancer	prognosis, infiltration of immune cells in TME	[114]
	scoring	bladder cancer	prognosis, infiltration of immune cells in TME	[138]
	scoring	gastric cancer	prognosis, infiltration of immune cells in TME	[139]
	scoring	cervical cancer	prognosis, infiltration of immune cells in TME	[140]
m6A/m5C/m1A	scoring	hepatocellular carcinoma	prognosis, infiltration of immune cells in TME	[141]
combined	scoring	prostate cancer	prognosis, infiltration of immune cells in TME	[142]
	scoring	glioblastoma	prognosis, infiltration of immune cells in TME	[143]

PUS1: pseudouridine synthase 1; PUS7: pseudouridine synthase 7; ADAR1: adenosine deaminase acting on RNA; m6A: N6-methyladenosine; m1A: N1-methyladenosine; m5C: 5-methylcytidine; m7G: N7-methylguanosine; TME: tumor microenvironment; WDR4: WD repeat-containing protein

**Table 2:** RNA modification inhibitors as therapeutic targets

RNA modification	target	inhibitor	function	cancer type	references
m6A	FTO	rhein	anti-multidrug resistance	acute myeloid leukemia	[144]
		MO-I-500	inhibition of cell growth	breast cancer	[145]
		R-2HG	inhibition of cell growth	leukemia and glioma	[146]
		meclofenamic acid	restoration of gefinitib sensitivity	non-small cell lung cancer	[147]
		FB23/FB23-2	promotion of differentiation	acute myeloid leukemia	[148]
		CS1/CS2	inhibition of cell growth and immune evasion	acute myeloid leukemia	[93]
		Dac51	promotion of T cell response and response to anti-PD-1 therapy	melanoma	[149]
		18,097	inhibition of cell growth and lung colonization	breast cancer	[150]
		FTO-43	inhibition of cell growth	glioblastoma, acute myeloid leukemia, and gastric cancer	[151]
		C6	inhibition of cell growth	esophageal cancer	[152]
	ALKBH5	ALK-04	inhibition of the infiltration of Treg cells and MDSCs, enhancement of response to anti-PD-1 therapy	melanoma	[78]
	METTL3	STM2457	inhibition of cell growth	acute myeloid leukemia	[153]
		UZH1a	inhibition of cell growth	acute myeloid leukemia	[154]
	YTHDF1	engineered small extracellular vesicles	promotion of IFN-γresponse, MHC I expression	gastric cancer	[155]
	IGF2BP1	small-molecule cucurbitacin B (CuB)	CD4+, CD8 + T cells, CD56 + NK cells, and F4/80 + macrophage infiltration	hepatocellular carcinoma	[117]
m1A	TRMT6 and TRMT61A	Thiram	inhibition of cell growth	hepatocellular carcinoma	[156]
Ψ	PUS7	NSC107512	suppression of tumorigenesis	glioblastoma	[62]
	DKC1	pyrazofurin	inhibition of cell growth	colorectal cancer	[157]

m6A: N6-methyladenosine; FTO: fat mass and obesity-associated protein; ALKBH5: AlkB homolog 5; MDSC: myeloid-derived suppressor cells; METTL3: methyltransferase-like 3; YTHDF1: YTH domain family; IGF2BP1: insulin-like growth factor 2 mRNA-binding protein; TRMT6: tRNA methyltransferase; PUS7: pseudouridine synthase 7

## Data Availability

No datasets were generated or analysed during the current study.

## Abbreviations

ADAR: Adenosine deaminase acting on RNA
ALKBH5: AlkB homolog 5
APC: Antigen-presenting cell
AZIN1: Antizyme inhibitor 1
CAF: Cancer-associated fibroblast
CDS: Coding sequence
cGAS: Cyclic GMP-AMP synthase
CTL: Cytotoxic T lymphocyte
CXCL9: C-X-C motif chemokine ligand 9
CXCR3: C-X-C motif chemokine receptor 3
eIF: Eukaryotic initiation factor
FXR: Fragile X-related
FTO: Fat mass and obesity-associated protein
G3BPs: GTPase activating protein (SH3 domain)-binding proteins
GPX4: Glutathione peroxidase 4
HNRNPC: Heterogeneous nuclear ribonucleoprotein C
IGF2BP: Insulin-like growth factor 2 mRNA-binding protein
IL: Interleukin
m1A: N1-methyladenosine
m1G: N1-methylguanosine
m22G: N2, N2-dimethylguanosine
m3C: 3-methylcytidine
m5C: 5-methylcytidine
m6A: N6-methyladenosine
m7G: N7-methylguanosine
METTL: Methyltransferase-like
MHC: I major histocompatibility complex I
NF-κB: Nuclear factor kappa-light-chain-enhancer of activated B cells
PD-1: Programmed death-1
PD-L1: Programmed cell death-ligand 1
PNI: Perineural invasion
Ψ: Pseudouridine
STAT: Signal transducer and activator of transcription
STING: Stimulator of interferon genes
TCR: T cell receptor
TNF-α: Tumor necrosis factor alpha
TRMT: TRNA methyltransferase
VEGFA: Vascular endothelial growth factor A
YTHDF: YTH domain family
ZCCHC4: Zinc-finger CCHC domain-containing protein 4
